# The ABC of BBA: A Personal Perspective of a 10 Point Plan

**DOI:** 10.1007/s00266-026-05734-1

**Published:** 2026-04-02

**Authors:** Donald A. Hudson

**Affiliations:** UCT Private Academic Hospital, Anzio Road, Observatory, Cape Town, South Africa

**Keywords:** Bilateral breast augmentation, Breast implant, Breast implantation, Breast

## Abstract

The aim of bilateral breast augmentation (BBA) is not just to enlarge the breast, but to create an *aesthetically* pleasing enlarged breast. This is an elective surgical procedure, often performed on young healthy women—yet the incidence of revision is in the region of 20%. There is a wide variation in what these patients want cosmetically. A “10 point” plan is presented which should be considered in every patient undergoing BBA. Each point addresses a critical issue in BBA. Understanding the *mechanics* of the procedure is as important as knowing the technical details of the operation. There is no simple “one operation for all” approach in breast augmentation, but the hybrid augmentation represents an advance in this procedure. The incidence of revision surgery should be lowered when a careful, “thought out” approach is adopted.

*Level of Evidence V* This journal requires that authors assign a level of evidence to each article. For a full description of these Evidence-Based Medicine ratings, please refer to the Table of Contents or the online Instructions to Authors www.springer.com/00266.

Bilateral breast augmentation (BBA) is one of the commonest cosmetic procedures. There is a lot of controversy regarding this procedure. There is also wide variation in what patients want [1–10]. There is also a difference regarding the ideal size and contour of a “perfect” breast [1–10]. Men and women perceive the “ideal” breast differently [1–10]. Also, different cultures want different things regarding the “ideal” breast [1–10]—although it appears most women want more projection.

This article does not address capsular contracture, nor ALCL, but rather tries to present principles and examines the mechanics of breast augmentation. Also, the advent of the hybrid breast augmentation suggests a paradigm shift in augmentation mammoplasty is occurring.

It is important to emphasize that the whole subject has been influenced by two important variables—the main one being that of achieving *adequate* soft tissue cover of the prosthesis—it is still pertinent and will, and should influence decision-making. This is a critical concept. The second issue, i.e. capsular contracture, previously the scourge of this procedure, is still relevant but not nearly as prevalent.

## Caveat 1: This Is “High Risk” Surgery

There is no similar procedure in cosmetic plastic surgery. The breast implant, a foreign body, which is placed into a “man made” surgical plane or space. The incidence of revision surgery is surprisingly high [11] for an elective procedure, not uncommonly performed in patients with little or, no comorbidities. There are a myriad of not uncommon complications, some of which are (partly) iatrogenic, including implant malposition, double bubble deformity, animation deformity, etc. This is in addition to surgery related complications, like haematoma, infection, etc. Also, breast asymmetry is not uncommon preoperatively in patients seeking augmentation [12].

One can (and should) point this out to the patient preoperatively, but they will still expect absolute perfection. Additionally, there may be asymmetry of the thorax, and preoperative scoliosis to compound these variables [13].

## Caveat 2: The Aim of the Plastic Surgeon Is Not to Just Enlarge the Breast

The aim of the plastic surgeon is to create an *aesthetically* enlarged breast. The ideal breast (in the west) has a distance of 21 cm from the suprasternal notch (SN) to the nipple [9, 10]. The distance from the nipple to the IMF is 7 cm—creating a ratio of 21:7. While there may be some debate regarding these figures and the perfect ratio, there is no doubt that the ratio exists. This ratio should always be maintained when the breast is augmented. It may become distorted when the IMF is lowered too much, as the distance from suprasternal notch to nipple does not change much with augmentation [4, 14, 15].

## Caveat 3: Understand the Mechanics of the Procedure

**Fig. 1:**
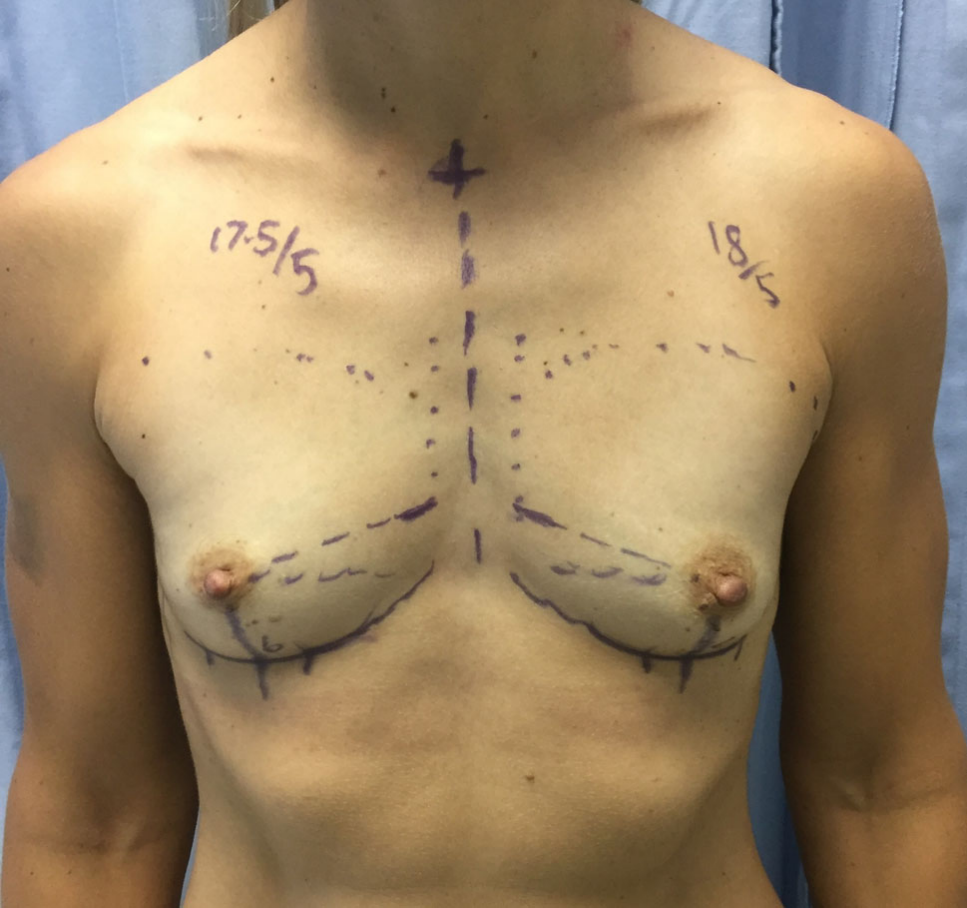
A thin muscular lady requesting breast augmentation. Note that the distance from the nipple to the medial border (radius, 7.2 cm in this patient) is longer than the distance from the nipple to the inframammary fold (5 cm in this patient). But, it is the breast base width (diameter, or 2× radius) which is used to determine to size of implant selected. In this patient, the breast radius measures 14.4 cm, whereas the distance from nipple to IMF is 5 cm (6 cm on stretch)

There are 2 key concepts to consider in breast augmentation; the first is that the centre of the implant *must* lie directly behind the nipple, (irrespective of the surgical plane chosen). The second is that the breast base width is critically important when selecting the prosthesis. However, the breast, particularly the lower pole is not a circular structure in the coronal plane. The distance from the nipple to the sternum (i.e. half the breast width or radius) is usually longer than the distance from the nipple to the IMF (Fig. 1). It is this latter measurement which usually needs to be lengthened. It needs to be lengthened such that the distance from nipple to IMF is equal to half the breast width (radius); this is to ensure that the centre of the prosthesis sits directly behind the nipple. In other words, the distance from the nipple to IMF should be similar in length to the radius of the prosthesis. Simplistically, this can be achieved by lowering the IMF—however there are 3 problems with doing this: firstly, this distorts the ratio of SN to nipple: nipple to IMF. In 20% of patients, the IMF is so poorly developed that it doesn’t matter and in another 20% the IMF is hypoplastic anyway [16], but the ratio still needs to be maintained. The second problem is that, if the IMF position is lowered to accommodate the prosthesis, the original IMF, a complex and unique anatomical structure [17] must be totally destroyed to prevent a double bubble deformity [17, 18] from occurring. Thirdly, the IMF must be meticulous reinforced and strengthened [19], as just the weight of the prosthesis will cause it to descend with time. Hall-Findlay et al. noted that the IMF was lowered about 2 cm [14] after breast augmentation.

The other important variable is the effect of the prosthesis on the tissues between the nipple and IMF, which often tends to be relatively taut. If this distance is not lengthened the prosthesis will be malpositioned. The distance from nipple to IMF will lengthen somewhat post augmentation. The force/pressure applied by the prosthesis will compress the breast tissue leading to tissue atrophy [20]. Also, tissue CREEP occurs due to tension and stretch, so the skin of the lower pole will expand and lengthen over time. The weight of the implant will also displace the IMF inferiorly [19]. As a result of these 3 forces, the distance from nipple to IMF will increase with time [4, 14] but this process takes a few months.

## Caveat 4: Which Plane/Pocket Yields the Most Natural Aesthetic Result?

From a purely cosmetic point of view, the most “natural” aesthetic result occur when the prosthesis is placed directly under the gland [21] (subglandular or subfascial). This provides a rationale for the more recent selection of this plane in breast reconstruction [21]. There is no breast tissue under the pectoralis muscle. The subfascial plane also preserves the Cooper ligaments [22]. Of course, not all patients want a “naturally enlarged breast.” Achieving adequate soft tissue cover of the prosthesis may also influence the decision regarding which plane to choose.

The total submuscle pocket had been superseded by the dual plane technique [23]. In fact, the total submuscular pocket was originally used in an endeavour to reduce capsular contracture [18]. It is important to remember that even in a dual plane technique, and the lower part of the prosthesis is *subglandular*. The dual plane provides upper pole fullness and increases soft tissue cover, but suffers the disadvantage of creating an animation deformity, and some reduction in pectoralis muscle power.

Dual plane 1 endeavours to minimise muscle power loss and provide more robust soft tissue cover. But, this occurs at the price of a greater animation deformity [13]. Also, the piston action of pectoralis major may displace the prosthesis both laterally and inferiorly [21]. This option has been discarded by a group with enormous experience in breast augmentation [13]. Their study highlighted the anatomical variations of pectoralis major and its impact on dual plane augmentation.

One advantage of placing the prosthesis submuscle is that it does tend to remain in a stable/fixed position as it is compressed and held in place by the muscle and is less vulnerable to changes in soft tissue occurring over time. However, with time and ageing, the weakened breast parenchyma may slide off the submuscular prosthesis, leading to a waterfall deformity.

## Caveat 5: The Inframammary Fold (MF)

It is important to emphasize the anatomy of the IMF [17, 18]. Firstly, the IMF extends from the medial aspect of the breast (sternum) to at least the anterior axillary line. In 25% of patients, the IMF is absent or rudimentary in patients seeking breast augmentation [16]. There are 2 macroscopic anatomical layers at the IMF divided by Scarpa’s fascia, viz. Above Scarpa’s fascia, there are well-defined fibrous bands between it and the skin. It is the more robust layer and will provide support to the prosthesis. In contract, the tissue between Scarpa’s fascia and the pectoralis fascia/muscle is more tenuous, and this plane is easy breached [18]. It is descent of the prosthesis in this plane that causes a double bubble deformity [18, 24].

Hence, it is postulated that in most cases, the original IMF should always be retained. The concept of lowering the IMF is obsoleteit harks back to the days when the breast width was ignored, and when only low profile prosthesis existed, and inserting a bigger prosthesis meant using a prosthesis with a larger diameter.

This lengthening of the nipple to IMF distance over a few months also emphasizes the importance of meticulous repair and fixation the IMF after using an IMF incision [19]. The weight of the prosthesis applies tension to the IMF and contributes to descent of the IMF over time.

## Caveat 6: Which Surgical Plane?

There is no ideal plane—that’s why there are various options. Again, achieving adequate soft tissue cover may affect this decision.

As already noted, the most “natural” aesthetic results occur when the prosthesis is inserted in a subglandular (or subfascial) plane. The problem is that many women wanting augmentation are very thin—and the subglandular plane in these patients carries the risk of prosthetic rippling and maybe visible and/or easily palpable, manifesting in both the lower and upper poles. This is where hybrid augmentation [25] may partially circumvent these problems from occurring (see also caveat 9).

The total submuscular plane is now essentially obsolete and has been replaced by the dual plane [23]. The dual plane concept is in some ways theoretically appealing. It offers the potential benefits of better soft tissue cover and lower pole expansion. But dual plane I, promoted for use in young thin nulliparous patients [23], in particular, has a few problems: firstly, the prosthesis maybe displaced by pectoralis muscle action to a more superior and lateral position [21]. In some patients, achieving an aesthetic cleavage maybe more difficult, and of course, the animation deformity is entirely un-aesthetic. More appealing is dual plane ii and iii—there is less of an animation deformity [13]. The superior edge of the prosthesis would not be visible in the very thin patient. Another huge advantage is the accentuation of upper pole fullness—while not “natural,” was commonly desired by patients seeking this operation. It must be borne in mind that any dual plane procedure has been shown impair muscle function, this is part of the tradeoff of dual plane augmentation. Hence, it is postulated that a dual plane iii maybe a better option [18]—upper pole cover of the prosthesis is provided with little animation deformity—but at the cost of some impairment of pectoralis power—so this may not a good option in body builders, etc.

The subfascial plane offers the theoretical advantages of a subglandular augmentation but also preserves Cooper’s ligaments [22] and may offer a more precise pocket to control implant position, but the fascia may limit skin envelope stretch [22, 26].

## Caveat 7: Which Incision?

Choose the access incision that provides the greatest chance of success, i.e. gives the surgeon the greatest degree of exposure and access to (any) of the surgical pockets and limits the risk of capsular contracture [14]. The best surgical access incision also limits the risk of asymmetry and implant malposition. Redo augmentation [11] is already in the region of 20%—which is too high for an elective preplanned operation.

It is important when planning a subglandular augmentation to ensure that excessive medial dissection does not occur, which would result in symmastia and require revision. Similarly, in a dual plane dissection, inadequate medial dissection may result in an excessively wide cleavage, also requiring revision.

A pocket that is too small for the prosthesis or a pocket that is too big for the prosthesis will result in the need for revision.

It appears that an IMF incision offers many advantages [15]. Additionally, there are anatomical variations of the pectoralis major muscle [13] (often under emphasized in the literature) which cannot really be discerned preoperatively, and an IMF incision allows this muscle to be adequately visualized.

## Caveat 8: Anatomical versus Round Prosthesis

This is another ongoing debate with no finite answer. There are proponents of both schools—which suggest that both options should always be borne in mind, and there are clinical situations where both options should be considered.

A number of articles have showed that even experienced surgeons cannot tell which type of prosthesis was used [27, 28]. The round prosthesis cannot rotate or flip. If the latter occurs, it means reoperation. It is tautological to do everything to reduce the risk of revision surgery.

Simplistically, round, higher profile prosthesis are chosen if the lady wants upper pole fullness and projection, whereas anatomical prosthesis will yield a more “natural shape.”

## Caveat 9: The Hybrid Breast Augmentation [25]

This combines autologous fat grafting (AFG) and an implant should now be considered the gold standard technique of augmentation. Thus, AFG offers a method to increase soft tissue cover of the prosthesis and improve aesthetics.

This means that the plastic surgeon should consider the importance of AFG in the primary augmentation, and not reserve it for “touch ups” and correction of wrinkling, etc. The breast prosthesis, particularly higher profile prosthesis provides projection to the breast—which cannot really be achieved with AFG alone. On the other hand, in a very thin patient with a wide intermammary distance, the only way to narrow the cleavage is with AFG.

As part of this approach, it allows the plastic surgeon to insert a *smaller* prosthesis. There are a number of problems using larger prosthesis. These include tissue atrophy if the prosthesis is  > 375 ml [29]. Additionally, a large prosthesis may require lowering of the IMF, which distorts the golden ratio mentioned above—thereby impairing breast aesthetics. A prosthesis which is too large predisposes to a double bubble deformity. It is important to bear in mind that breast prosthesis is also quite heavy (a 300-ml prosthesis weighs 312 g). Hence, large prosthesis applies a greater downward force on the IMF and will displace it inferiorly irrespective of how well the IMF is fixed after prosthetic insertion. Hence, there are cogent reasons to adopt this approach.

## Caveat 10: The Long Term “Complications” Are Proportional to the Size of the Prosthesis Chosen [2, 29, 30]

Irrespective of the surgical plane used, it must be borne in mind that this is not a “natural” cavity and any foreign body (prosthesis) will apply pressure to the surrounding tissue. The breast tissue is softer and will bear the brunt of this [20, 29]. In other words, a large prosthesis will cause some pressure necrosis of the breast tissue leading to a reduction in breast tissue over time [20, 29]. The degree to which this occurs is variable but a larger/profile prosthesis clearly applies more pressure. The submuscular plane may partly absorb some of this pressure, but pressure necrosis will still occur.

Additionally, the weight of the prosthesis will tend to displace the prosthesis inferiorly. The bigger (and therefore heavier) the prothesis, the greater this force on the IMF—this is the basis of Hooke’s law [30]. Some investigators have even tried reducing the weight of the prosthesis [30].

It is interesting to note that generally, there appears to be a shift towards inserting smaller prosthesis [31].

Breast augmentation is often perceived as an easy and quick procedure. However, complications both short term and long term, are not uncommon. As noted above, BBA is unique as a cosmetic procedure. There is no “one size fits all approach.” Careful planning and thought is required to lead to consistent results. The rate of surgical revision needs to be lowered.
